# Wearable Devices Based on Bioimpedance Test in Heart-Failure: Design Issues

**DOI:** 10.31083/j.rcm2509320

**Published:** 2024-09-09

**Authors:** Santiago F. Scagliusi, Luis Giménez-Miranda, Pablo Pérez-García, Alberto Olmo-Fernández, Gloria Huertas-Sánchez, Francisco J. Medrano-Ortega, Alberto Yúfera-García

**Affiliations:** ^1^Institute of Microelectronics of Seville - Spanish National Center of Microelectronics (IMSE-CNM) University of Seville, 41092 Seville, Spain; ^2^Institute of Biomedicine of Seville (IBiS-US), Hospital Universitario Virgen del Rocío (HUVR) University of Seville, 41013 Seville, Spain

**Keywords:** bioimpedance, electrical impedance, wearable devices, heart failure, electrocardiography, bioimpedance vector analysis, total body water, internet of things

## Abstract

Heart-failure (HF) is a severe medical condition. Physicians need new tools to monitor the health status of their HF patients outside the hospital or medical supervision areas, to better know the evolution of their patients’ main biomarker values, necessary to evaluate their health status. Bioimpedance (BI) represents a good technology for sensing physiological variables and processes on the human body. BI is a non-expensive and non-invasive technique for sensing a wide variety of physiological parameters, easy to be implemented on biomedical portable systems, also called “wearable devices”. In this systematic review, we address the most important specifications of wearable devices based on BI used in HF real-time monitoring and how they must be designed and implemented from a practical and medical point of view. The following areas will be analyzed: the main applications of BI in heart failure, the sensing technique and impedance specifications to be met, the electrode selection, portability of wearable devices: size and weight (and comfort), the communication requests and the power consumption (autonomy). The different approaches followed by biomedical engineering and clinical teams at bibliography will be described and summarized in the paper, together with results derived from the projects and the main challenges found today.

## 1. Introduction 

Wearable sensor devices are importantly transforming current healthcare by enabling the non-invasive and continuous monitoring of patients’ biomarkers or physiological signals. There are different medical areas where wearable devices are currently being used by physicians, such as in rehabilitation or home monitoring of elderly people. However, there are other areas where sensor devices are not robust or precise enough yet for physiological monitoring.

In cardiology, the electrocardiography (ECG) signal, measuring the electrical activity in the heart, has traditionally been the most studied signal. However, there are different parameters important in heart failure, such as cardiac output or arterial blood pressure, which cannot be well studied with only ECGs [1]. It is necessary to use other technologies that enable more detailed analysis to study the progression of heart-failure patients.

Impedance spectroscopy has long been used for monitoring a wide variety of important medical parameters and biological signals [2]. In cardiology-related diseases, impedance spectroscopy has received important attention in recent years, as several methods have been proposed for cardiac monitoring, determining hemodynamics or predicting future cardiac risks, among others [2]. In heart failure diagnostic and control applications, the use of impedance spectroscopy to monitor the patient volume status and to detect edema is a recent active research area. The use of impedance spectroscopy applications in cardiology-related diseases can bring important benefits for the remote monitoring of heart failure patients, in an affordable and non-invasive way.

However, despite the high number of research articles in the use of impedance spectroscopy for cardiology-related diseases and heart failure, only a few devices have successfully reached commercialization and use in clinical operation [3]. Different reasons can be found, among which we find the lack of complete clinical validation of studies and, in relation with it, the lack of reproducibility of results and robustness of measurements [4]. Furthermore, these devices are not designed as wearable devices, and do not allow the continuous monitoring of physiological parameters. There are still different engineering challenges for the wide adoption of clinical devices that will be studied in the present work.

In this review, we study in a systematic way the different applications found for electrical impedance in cardiac afflictions and heart failure. We describe the technique of impedance spectroscopy from its physical fundamentals, and study the current engineering challenges of the technology, from the design of the electrodes to the impedance monitoring system, including engineering challenges such as wireless communications or power requirements. This study helps us analyse the limitations in the design and implementation of medical devices for its use in clinical environments, and shows us the future roadmap towards the implementation of clinical wearable devices for the monitoring of heart failure patients.

## 2. Materials and Methods

### 2.1 Search Strategy and Selection Criteria

We searched for scientific works that researched into the use of wearable devices in heart failure, which were published between 2000 and 2023. Google scholar database was used. The search included the following key words: “heart failure” and “wearable devices”, and “electrical impedance”, and “bioimpedance”.

We divided our review in different sections, and studied different aspects of the use of wearable devices in heart failure, from the more generic aspects of the clinical use of impedance measurement in heart failure, to the physical aspects of the sensing technology, and finally to the engineering aspects of these devices.

### 2.2 Inclusion Criteria and Data Analysis

We included in our analysis only those studies that met the following criteria: (1) they were published between 2000 and 2023, (2) they were published in English, and (3) they are suitable to answer the main research questions for the different sections:

∙ Electrical impedance in heart failure: What are the main uses of electrical impedance in heart failure?

∙ The sensing technique and impedance specifications: What are the main requirements for the correct use of impedance sensing in biological material?

∙ Electrode selection: What are the main requirements of electrodes for its use in electrical impedance applications?

∙ Bioimpedance (BI)-wearable devices: portability and comfort: What are the main requirements of patients for the use of wearable devices in electrical impedance applications?

∙ Wireless communication and power consumption: What are the main engineering constraints and challenges found in the wireless communication of data and power consumption in electrical impedance applications?

## 3. Results and Discussion

109 articles were identified, of which 3 were removed before screening because of duplication of results. Of the 106 remaining articles, 39 of them were excluded by reviewing the title and summary. The main reasons were that these works focused on other medical issues not clearly related with heart failure, such as respiratory, renal or musculoskeletal conditions, or that they focused on an engineering area not clearly related with bioimpedance measurements.

The remaining 67 articles were classified following the aforementioned research questions in the following categories: electrical impedance applications in heart failure (23 articles), the sensing technique and impedance specifications (8 articles), the electrode selection (9 articles), BI-wearable devices, portability and comfort (18 articles) and wireless communication and power consumption (autonomy) (9 articles).

**Fig. 1.**
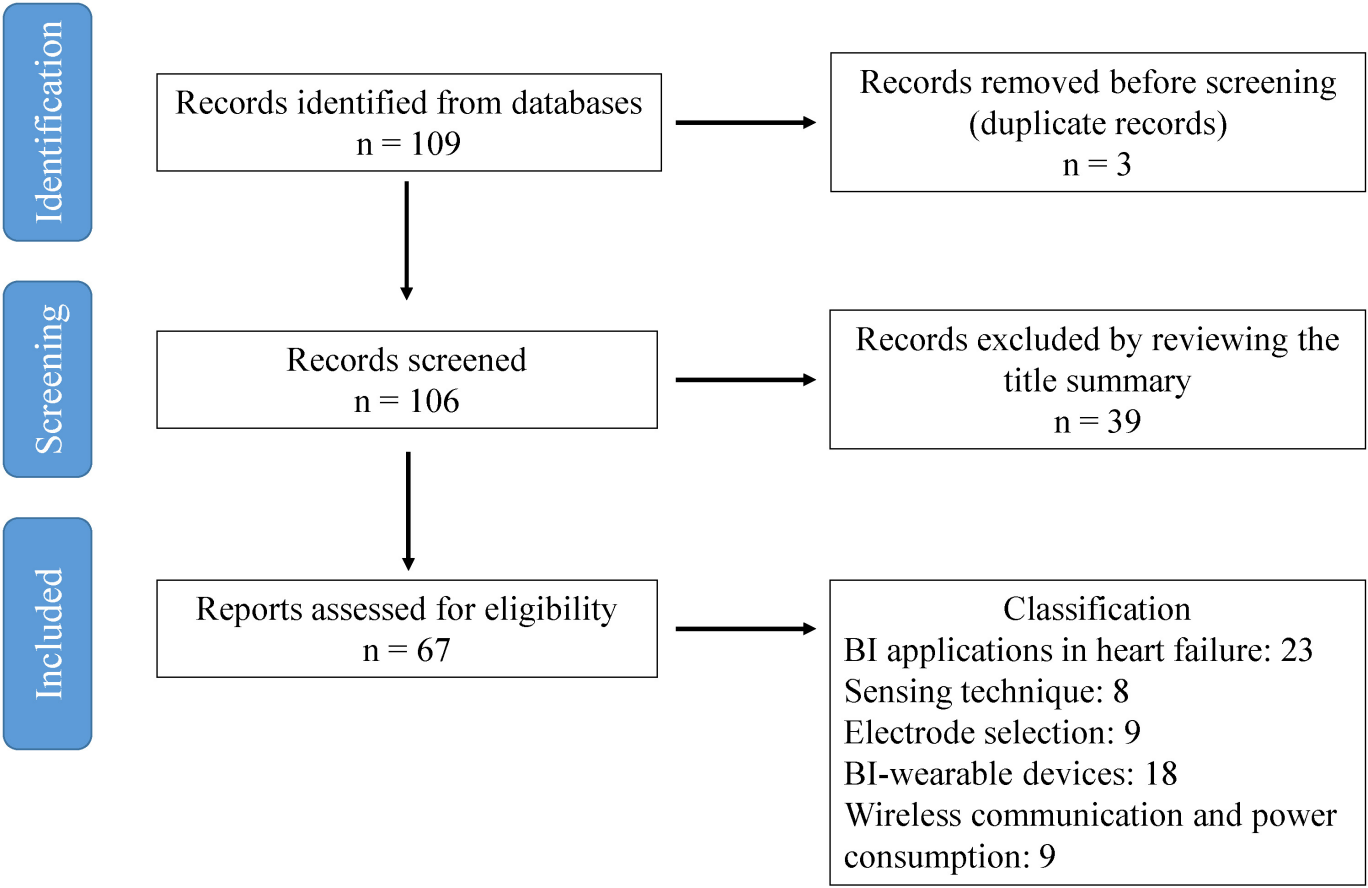
**Flow diagram for identifying eligible articles**. BI, bioimpedance.

The flow diagram followed for identifying eligible articles is shown in Fig. 1.

Additional references were included in the different sections, in order to better describe the fundamentals of impedance sensing, deeply understand the problem of electrode selection, and analysing the remaining engineering challenges in the wireless communications and power consumption of these devices, as it will be shown in the following sections.

### 3.1 Impedance Measurement Applications in Heart Failure

Bioimpedance is the name that receives the measurement of electrical impedance in biological material. To implement it, electric current flows through the body, and the voltage is measured to calculate its relation, which receives the name of impedance (being this magnitude composed of resistance and reactance).

We used the following main categories for current applications of impedance measurement in heart failure, according to the different works found in our systematic revision:

∙ Assessing volume status in heart failure patients: The detection of edema is an important factor in the prognosis of heart failure [2, 5, 6]. As electrical impedance depends on tissue composition, this technique has been widely used to estimate the degree of edema formation in heart failure patients [5, 6, 7, 8, 9]. Bioimpedance vector analysis (BIVA) is another variation of electrical impedance measurements that has received an important attention in heart failure [10]. With this technique, the real part and imaginary part of impedance are shown for a specific frequency (50 kHz in most cases). Different elliptic regions were modelled to identify differences in hydration status and body composition components [11]. The technique has proved to efficiently detect peripheral congestion in acute decompensated (ADHF) and chronic heart failure (CHF) [12]. The published scientific evidence on this technique has led to its proposal as a score for clinical practice in heart failure patients [13].

∙ Monitoring of hemodynamics: Monitoring hemodynamics provides additional information about the patient’s clinical condition [2, 14]. Events in the cardiac cycle are correlated to their position on the impedance signal [3, 15]. The impedance signal can also help assess cardiac function, output and stroke volume [16]. Ambulatory impedance cardiography was used for hemodynamic monitoring during the activities of daily living [17]. Bioreactance is also based on electrical impedance, but it is focused on the electrical resistive, capacitive and inductive properties of blood and biological tissues inducing phase shifts between the applied electrical current and the resulting voltage signal [18, 19, 20]. These phase shifts are used to study instantaneous physiological changes, such as aortic flow [18], and represent in these cases an accurate tool to analyse cardiac output.

∙ Integration with other signals for cardiac monitoring: other biosignals have also been studied for cardiac monitoring. Cardiac output or arterial blood pressure, important in heart failure, cannot be well studied only with ECG [1]. Electrical cardioversion has been proposed for cardiac rhythm control [21]. Wearable ballistocardiogram and seismocardiogram-based systems for monitoring relative changes in cardiac output, contractility, and blood pressure was proposed in [22]. These signals can also be used in conjunction with electrical impedance for cardiac monitoring. For example, a system based on multiple parameters (thoracic impedance, heart rate, electrocardiogram and motion activity) has been proposed in recent articles [14].

∙ Prediction of future risks in heart failure: Electrical impedance has also been proposed as a direct method to estimate and predict decompensations in heart failure patients [23, 24]. In different works, intrathoracic impedance monitoring was used to predict decompensated heart failure and provide early warning preceding hospitalization [25, 26, 27]. Changes in monitored impedance of patients can be correlated with patient outcomes on rehospitalization, decompensation, and mortality [28].

The use of artificial intelligence algorithms is also receiving increasing interest in medical areas over the last few years [29]. The use of machine learning strategies with different monitoring devices for cardiovascular management is described in [30]. A new heart function index—a composite algorithm of non-invasive hemodynamic biomarkers from a cardiac scale—in predicting worsening heart failure (HF) events is proposed in [31], based on a prospective and multicenter study. Finally, according to [4], remote monitoring (through accurate and reliable signals, and personalized algorithms) should be accompanied by a system that engages, informs and empowers patients, for an efficient home management of heart failure.

A common conclusion in the previous described works is the need to have highly reproducible methods for clinical practice, with robust and stable signals, and the support of large clinical studies, before its introduction in the clinical practice. BIVA has been proposed for clinical guidelines [13] but in many other cases, the reported prototypes have not passed through complete clinical validation. In order to study the reasons for this, we must study the fundamentals of the technique, as it is shown in the following sections.

### 3.2 The Sensing Technique and Impedance Specifications

To design an efficient method to measure bioimpedance in biological materials, two parameters must be first taken into account: the range of impedance values to be measured, and the frequency spectrum needed for measurements [32]. These are the main system specifications that need to be taken into account when designing bioimpedance monitoring circuits.

Electric models for electrodes and body BI (tissue, organs, etc.) are crucial for a correct interpretation of the data measurement from the body [33, 34, 35]. Different works can be found with different models, many of them based on [36]. These models are used in the final decoding of impedance measurements, after comparing with the experimental measurements. These models can be focused on single cells, or, more commonly, tissues or body sections [6].

To measure any bioimpedance Z_x_, with magnitude Z_a⁢b⁢s_ and phase ϕ (or real part and imaginary part) it is necessary to provide an AC signal, with ω as the input signal frequency, which is used to excite the sample under test, Z_x_. The response (either current or voltage) is usually measured after a voltage amplifier or a trans-impedance amplifier. The useful information (Z_a⁢b⁢s_, ϕ) is obtained after the processing of these measurements [6]. BIVA consists of the specific measurement of the real part and imaginary part at a specific frequency [10]. Bioreactance is another term used for the specific application of bioimpedance to study phase shifts between the applied electric current and obtained voltage [18]. In our article we have studied bioimpedance measurements from a more generic point of view.

**Fig. 2.**
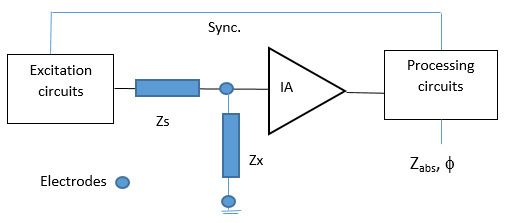
**General block diagram for the measurement of impedance**. Impedances Zx and Zs are found between the excitation circuit and the Instrumentation Amplifier (IA). Final impedance Zabs and phase ϕ are obtained by the processing circuit. Based on [39].

Excitation and processing circuits are required in bioimpedance applications [33]. Excitation is usually carried out with AC current sources, being the mission of the circuit to decode the voltage response to signal excitation, to finally determine the impedance of the biological material. In most cases it is necessary to establish a synchronization with the ac excitation circuits [6]. Classical approaches consist on coherent demodulation principle [37] or synchronous sampling [38], which in general result in good performances. A general block diagram of the general processing circuit is illustrated in Fig. 2 (Ref. [39]).

In several applications [40], a simple solution is proposed based on a resistance to recover the in-phase reference signal to extract the full impedance components of Z_x_. Another implementation is the use of a microprocessor to minimize the analogue part of the signal processing circuits [15, 41, 42, 43, 44], where this approach is used to monitor body fluid composition.

Maximum impedance values reported in Total Body Water (TBW) test are in the range for 250 to 1000 Ω [45] from several body sections, being reduced to 10 to 80 Ω in HF test performed in chest [28] and legs [6]. Frequencies of interest go from 3 kHz to 1 MHz [45, 46], depending on the biological material. In HF applications where the water content (extracellular and intracellular) is measured, the most common frequency range used varies from 1 kHz (low frequency) to 200 kHz (high frequency) [47]. The 50 kHz frequency is the most accepted frequency value for a single frequency (one-shot) test [28].

Clinical bioimpedance applications in heart failure are based on commercial equipment, which are not useful for wearable systems, mainly because of the large size of the equipment used. Examples can be found in Impedimed (SFB7) [48], Medtronics (Optivol) [49] or RJL Systems (Quantum/S Analyzer) [50]. Some approaches have tried to evolve toward portable devices [51], but they are not comfortable enough for continuous monitoring of BI, because of the use of wet electrodes, as it will be shown in the following sections.

### 3.3 The Electrode Selection

Electrodes are the interfaces responsible for converting electric currents from a conductive metal, part of the measurement system, to the biological material (human body in the case of heart failure) [6]. They are one of the most crucial parts in the monitoring system, and a special focus should be put on the design of them.

The most common type of electrode used in cardiac clinical applications has been the Ag/AgCl electrode. There are several reasons for this, such as their stability and non-polarizability. However, several drawbacks can also be found, such as their deterioration after a few hours of continuous use [52].

In recent years, dry electrodes have been studied as an interesting alternative to Ag/AgCls electrodes, being deemed more appropriate for its use in wearable devices, as they can be used for long periods of time, without affecting the skin. In this context, electrodes made of stainless steel have been reported to work well for bioimpedance spectroscopy [24, 53, 54].

The standardization of the electrodes required to measure bioelectrical parameters in vivo, have not been well defined yet. For example, there is a lack of agreement regarding the best option in the fabrication materials used for the electrode [52]. Different physical properties must be taken into account when selecting the right electrode for bioimpedance measurements [6]:

∙ Accuracy: low impedance in the electrode-skin interface is advisable, to improve the measured signal-to-noise ratio.

∙ Stability: the measurements must be stable, and not time dependent [55]. This is of special importance in heart failure applications, where the evolution of the patient is being monitored.

∙ Non-toxicity: in order to avoid unwanted secondary effects in the patient’s skin.

∙ Geometry: depending on the specific application and the nature of the biological material to be measured.

In principle, taking these points into account, dry electrodes are better electrodes for commercial wearable devices [53, 54]. However, compared to Ag/AgCl electrodes, their signal-to-noise ratio is worse, because of a higher input impedance, and high input impedance amplifier must also be used in the monitoring circuit [56].

**Fig. 3.**
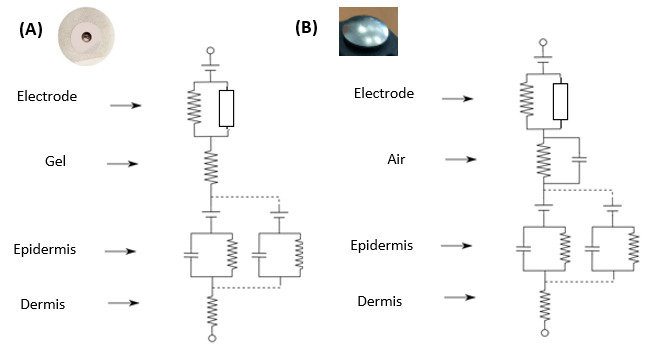
**Electrical models of skin-electrode interfaces according to material**. (A) Ag/AgCl (gel), and (B) dry stainless steel electrode (air) (based on [57]).

The equivalent electrical models of both types of electrodes (Ag/AgCl and dry electrodes) can be seen in Fig. 3 (adapted from [57]), where the electrical modelling of each layer in the interface between the skin (dermis) and the electrode can be observed. A good permanent contact with the skin is also necessary for dry electrodes, and this one of the most difficult challenges faced in wearable devices [58].

Research on textile electrodes has also received interest in recent years [59, 60, 61]. Textile electrodes are presented as a very good option for the implementation of wearable devices, which can achieve the successful monitoring of impedance in heart failure patients, for long periods of time, in a non-invasive and comfortable way. More recently, the use of nanotechnology has been presented for the estimation of edema and swelling, with a stretchable sensor fabricated with a combination of a polymer, carbon nanotubes and silver nanoparticles [62]. The use of small-sized distributed smart bio-impedance patches is also proposed in [63], as another similar solution for bioimpedance monitoring.

### 3.4 BI-Wearable Devices: Portability and Comfort

Wearable devices are designed to be used during long periods of time, and need to fulfil important requirements in terms of comfort. A design must be followed with reduced dimensions, reduced weight, and integrated where possible with daily used garments. These requirements are even more important with heart failure patients, as it is a medical condition often associated with people of advanced age.

The importance of a small size and low weight in monitoring devices for congestive heart failure patient has been studied [51], showing the need to avoid discomfort to patients. The need for the right electrodes to minimize skin damage in clinical settings has also been studied [64].

Some of the better examples of integration of electrodes in daily garments can be found in children clothing [15] or sport applications [65]. The importance of this integration on the user comfort has been well described in a wide variety of applications [15, 61, 66]. However, textile electrodes can bring other problems, such as the deterioration of the electrode, due to a number of reasons, including the presence of dust and contamination [67].

Autonomy of the wearable device is also related with portability and comfort, as the size of batteries often limit the reduced size required for the monitoring device. Autonomy in wearable devices is directly related with the energy required for the operation of the circuits, wireless communication, and data transmission [6]. The next section (3.5) will further describe some issues about this topic.

There are other more recent alternatives to the use of textile electrodes that can also increase the portability and comfort of bioimpedance sensing applications. The use of small-sized distributed smart bioimpedance patches is proposed in [63], where the communication between the patches is established through the human body, eliminating the need for electrical wires. Organic ultrathin devices are also being proposed and tested for their ability to adhere to the skin, similar to a temporary-transfer tattoo [68]. Body sensor networks, including several sensor modalities for measuring vital signs have been studied [69], assessing the comfort of different positions of the electronic components. Elderly patients were found to be a challenging target group, as dressing is often a major issue for them.

### 3.5 Wireless Communication and Power Consumption. Engineering Challenges

Wireless communication and power consumption are two important engineering requirements, which in many cases affect other characteristics previously seen, such as portability and comfort. The Internet of Things (IoT) paradigm has been applied to many different scenarios [70]. This paradigm is also currently receiving important attention in clinical settings, where two main different scenarios are being reported. In a first scenario, the patient is on the hospital premises, under medical surveillance. In this case, a data gateway device could be placed in the patient’s room, and standard technologies such as Bluetooth [71, 72] could be used to communicate the data from the sensor device.

In the second scenario, the patient is outside the hospital, remotely-controlled by the team of physicians. In this case, the use of smartphones is usually followed by prototype applications, also using standard technologies such as Bluetooth to communicate the sensor device with the patient’s smartphone, for example [72]. This is an especially interesting case for heart failure patients, as the progression of the patients’disease can be remotely monitored by physicians, triggering different alarms when, for example, the edema in the patient rises higher than determined thresholds [6].

**Fig. 4.**
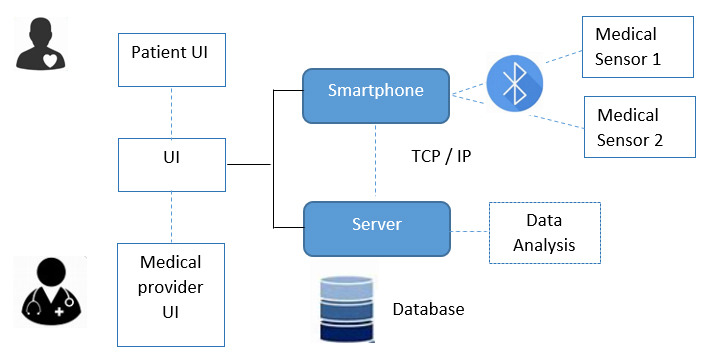
**Block diagram for the Wireless Communication via Bluetooth (Device/Smartphone) and TCP/IP (Smartphone/Server)**. Different User Interfaces (UI) are provided in the system for patient and medical providers. The protocol TCP/IP (Transmission Control Protocol/Internet Protocol) is commonly used (based on [6]).

A typical architecture proposed for the second IoT scenario is shown in Fig. 4 (Ref. [6]). In this figure, different medical devices worn by the patient (like the bioimpedance sensor) are connected via technologies such as Bluetooth to the patient’s smartphone. Data is transferred via internet to the medical server, where it is stored and further processed and analysed by the physician.

Finally, power consumption is a key element in the design of medical wearable devices. Several studies focus on the importance of the right choice of wireless technology (such as Bluetooth Low Energy or Bluetooth 5) in the general power efficiency of the application [28, 73]. However, the amount of data recorded in bioimpedance applications has important implications on the design of these monitoring systems [74]. Even though real-time applications are not generally required in heart failure clinical applications, it is usually necessary to monitor patients’ data for long periods of time. If different frequencies are used and short periods of time for acquisition are required, this can lead to an important magnitude of acquired data. Based on previous experiences [6], for edema monitoring, an acquisition time of hours is reasonable to explain the daily evolution of fluid accumulation in patients.

Energy consumption is a common complication in wearable computing in general and to activity monitoring in particular. Wearable devices must consider an autonomous power supply source delivering the energy required for the correct work of all electronic circuits. In contrast to implantable electronics, such as pacemakers, this energy source can be easily changed when required. Lithium batteries are generally employed for powering wearable devices due to its excellent properties: small weight (around 0.5 g/cm^3^), stability level for voltage (–3.04 V), low output resistance and high electrochemical equivalent (0.259 g/Ah). To optimize the battery life for low and lower power consumption, several strategies can be followed:

∙ Design and adequate firmware. A specific and low sampling data rate can be enough to efficiently monitor the process in many cases.

∙ Use of specific circuits for energy manipulation (Power Management Unit, PMU).

∙ Design of Low-Power (LP) circuits to minimize supply voltage, circuit complexity, clock frequencies, direct current sources (DC) current sources values and capacitance of switching nodes.

∙ Use of a real time battery control, to enable the users to evaluate the battery level from the application in its cellular phone.

As an example, energy management on wearable devices, the NRF52832 microcontroller is employed in [60], being the schematic based on the internal DC/DC regulator setup, reducing power consumption to a minimum (3.7 mA with the CPU running and 1.2 µA in sleep mode). A low power architecture for impedance measurement was proposed in [75], reducing the consumption in the most critical blocks of the system: the current driver, the signal sensing and the demodulator. As another interesting approach to avoid the problem of power consumption, self-powered electronic devices are proposed in [76], harvesting energy from the body and its ambient environment.

### 3.6 Economic and Social Considerations

Finally, the potential use of bioimpedance wearable devices in clinical settings for heart failure has important societal and economic considerations that are well worth mentioning.

As in other telehealth studies, different reports show the potential improvement in the health of heart failure patients[4]. The technique could be used in remote monitoring, for both home and hospital environments. According to this study, the remote monitoring of the patient, in addition with a system of care that engages, informs, and empowers patients, which is essential for the effective home management of heart failure, in order to control symptoms and avoid the re-hospitalization of patients. It can also be an interesting tool in hospital environments where clinical resources are scarce, provided there is efficient management of the information, using impedance data as an additional support tool, and not as a substitution for clinical operation [4].

Economic aspects should also be taken into account. The assessment of the economic impact and clinical consequences of telehealth in chronic heart failure was also studied in [77]. In this study, a Markov model was developed in the context of a home-based telehealth program on chronic health failure, with important cost savings for heart failure patients in the range from 1 year to 5 years. In general, the potential of telehealth solutions for the reduction of economic costs for healthcare systems is well supported by scientific literature [78].

## 4. Conclusions

Bioimpedance represents a useful technology for the monitoring of clinical parameters in heart failure patients. Bioimpedance measurements are mainly used in the following areas: assessing volume status of heart failure patients, monitoring hemodynamics, integrated with other monitoring cardiac signals, or as a predicting tool itself.

For heart failure wearable devices applications where long term measurements are expected, dry or non-contact electrodes are the best option to reduce skin damage [6]. Its electric model should be incorporated as design specifications for bioimpedance measurement circuits. Frequency range of interest varies from 1 to 100 kHz, with 50 kHz classed an intermediate relevant test point for an on-set BI test. Current heart failure bioimpedance systems must evolve towards more size-reduced versions, which are not burdensome to patients. The implementation of specific integrated circuits (ASIC), which will enable a reduced system size for a better patient comfort should be considered as a future objective. Reproducibility of measurements is a key factor for the adoption of these technologies into clinical practice.

We find one of the most important engineering challenges is the reproducibility of measurements and the wearability of the electrodes used. The invention of gel-free dry electrodes have led to a revolution in the field of healthcare monitoring, overcoming the limitations that wet Ag/AgCl electrodes had when implementing home remote sensing devices [53, 54, 77]. The integration of electrodes in textile garments has now provided unprecedented possibilities in healthcare monitoring. Textile electrodes have many advantages over traditional wet Ag/AgCl electrodes, such as being comfortable for the user. Wearable conductive textile electrodes have recently been proposed for monitoring biopotential signals [59, 79], and some of them have also been tested in heart failure applications. Remaining engineering challenges can be found in the reproducibility of results for clinical practice, the comfort and usability of the wearable device, and in its power consumption. The potential of these devices in the reduction of costs for public healthcare systems and the improvement of patients’ health has been outlined in this review.

## Abbreviations

BI, bioimpedance; WD, wearable devices; HF, Heart failure.
